# Recurrent Pectus Excavatum Repair via Ravitch Technique With Rib Locking Plates

**Published:** 2014-12-02

**Authors:** Chetan Pasrija, Brody Wehman, Devinder P. Singh, Bartley P. Griffith

**Affiliations:** ^a^Division of Cardiac Surgery, University of Maryland School of Medicine, Baltimore, Md; ^b^Division of Plastic Surgery, University of Maryland School of Medicine, Baltimore, Md

**Keywords:** pectus excavatum, rib prosthesis, rib locking plates, modified ravitch technique, recurrent pectus excavatum

## DESCRIPTION

A 39-year-old patient presented with dyspnea and right ventricular compression secondary to recurrent pectus excavatum (PE). The patient's concavity was surgically relieved. A ladder plate was longitudinally attached and 4 rib-locking reconstruction plates were placed. At 6 months postoperation, the patient was ambulating without dyspnea and imaging showed relief of the right ventricular compression.

## QUESTIONS

1. What is the etiology of pectus excavatum?
2. What signs and symptoms are related to this disorder?
3. What are the indications for surgery, and what are the common operative interventions?
4. What is the recurrence rate, and what factors are associated with recurrence?

## DISCUSSION

While PE has no known definitive etiology, it has been associated with both connective tissue defects and scoliosis. In one series, 19% of patients with PE had clinical features of Marfan's syndrome and in a separate study, 21% of patients had clinical and radiologic evidence of scoliosis.^1^^,^^2^ Pedigree analysis has shown that PE is an inherited disorder, often with Mendelian characteristics.^3^ However, as seen in this case, patients often present with isolated PE without a family history of this disease.

PE is often asymptomatic during childhood secondary to extensive pulmonary and cardiac reserve along with a pliable chest wall. However, as children grow, they become dyspneic and their exercise tolerance decreases significantly. As the chest wall becomes less pliable, the patient's ability to inspire becomes increasingly restricted. This was demonstrated clinically and diagnostically in our patient, who developed shortness of breath at rest requiring home oxygen and a restrictive physiology on pulmonary function tests (PFTs).

Evaluation for surgery includes computed tomographic scan of chest, PFTs, and a cardiac evaluation. Severe PE is considered a Haller Index (HI) greater than 3.25 (HI = ratio of transverse diameter to the anteroposterior diameter, normal = 2.5), PFTs indicative of airway disease, clinically relevant compression of the heart, or progression of disease with physical symptoms.^4^ Our patient had an HI of 3.5, restrictive disease on PFTs (forced vital capacity (FVC) = 46% predicted, forced expiratory volume in 1 second (FEV1)/FVC = 90%), and significant concavity of the sternum at the level of the third, fourth, and fifth ribs, which were impinging on the right ventricle. The most common surgical interventions are the Ravitch and Nuss procedures. The Ravitch, or “open,” procedure consists of resection of the concavity and associated subperichondrial cartilage followed by fixation of the sternum and reattachment of the ribs. The Nuss procedure, known as the minimally invasive approach, involves placing a custom round steel bar just behind the sternum and traversing to the pleural spaces to create outward pressure on the sternum at the point of concavity.

Recurrence of PE after surgical repair is not uncommon, present in approximately one-third of patients. Factors associated with recurrence include surgical technique, age of operation, infective processes, and genetic processes.^2^ Our patient initially underwent a modified Ravitch repair at the age of 4, but represented at the age of 39 with symptomatic PE. While the Ravitch technique and Nuss procedure have proven to be safe and effective means of repair after recurrence,^4^^,^^5^ multiple other strategies have been proposed.^5^^,^^6^ The cornerstone of these modifications is to increase sternal stability and thereby prevent recurrence. To stabilize and reconstruct the chest wall in this case, an 8-hole ladder plate was placed superficially for fixation of the transverse sternotomy. Then, the lower aspect of the sternum was secured with implantation of four 16- to 18-hole rib-locking reconstruction plates (Synthes, West Chester), 2 on each aspect of the fourth and fifth ribs. These extended back from the lateral edge of the ribs to the sternum, where 2 to 4 screws were placed in the ribs and sternum. Lateral chest wall stability was obtained along with sternal stability. Primary bilateral myocutaneous pectoral muscle flaps were secured to provide durable coverage of the reconstruction plates.

With this case, we present a new alternative for patients with complex, recurrent PE.

## Figures and Tables

**Figure 1:**
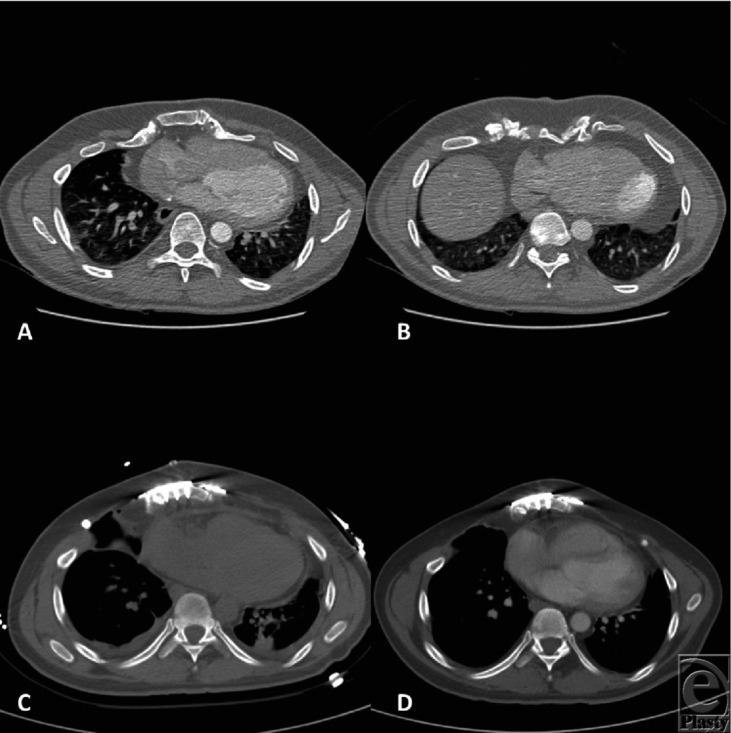
(*a* and *b*) Preoperation computed tomography showing concavity. (*c*) Postoperative at 5 days, showing relief of RV pinch. (*d*) At 4 months, there continues to be relief of RV pinch. There is also remodeling of heart, allowing for greater left lung capacity. RV indicates right ventricle.

**Figure 2:**
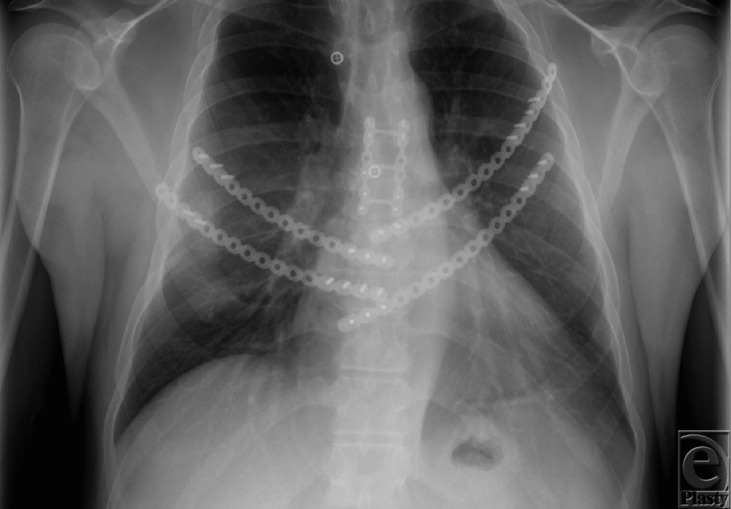
Postoperative posteroanterior chest roentgenogram. Notice the artificial rib technology at the lateral edge of the ribs and midline at the sternum. Also, the ladder plate providing sternal stability for the transverse sternotomy.

**Figure 3:**
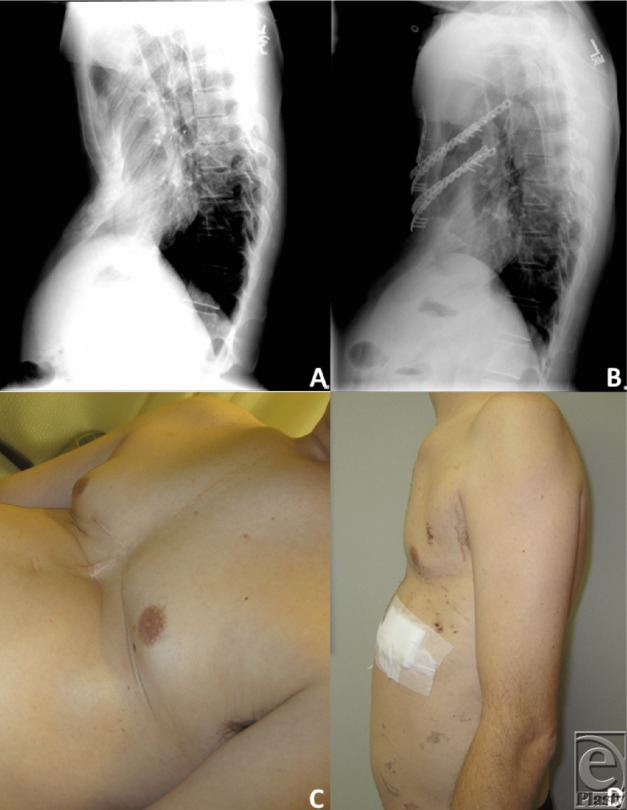
Preoperative (*a*) and postoperative (*b*) roentgenograms, showing change in concavity with the ladder plate and artificial ribs in place. Preoperative (*c*) and postoperative (*d*) patient images showing cosmetic and structural improvement.
